# Profile of Invasive Fungal Infections in a Paediatric Haemato-Oncology Unit: A Five-Year Retrospective Cohort Study From a Tertiary Care Centre in South India

**DOI:** 10.7759/cureus.116037

**Published:** 2026-09-10

**Authors:** Rishab Bhurat, Divya Nandakumar, Ashwini Harish, Latha Sneha, Padmasani Venkat Raman, Shanthi Mariappan, Rajoo Ramachandran

**Affiliations:** 1 Pediatric Oncology, Sri Ramachandra Institute of Higher Education and Research, Chennai, IND; 2 Pediatrics, Sri Ramachandra Institute of Higher Education and Research, Chennai, IND; 3 Pediatric Hematology and Oncology, Sri Ramachandra Institute of Higher Education and Research, Chennai, IND; 4 Microbiology, Sri Ramachandra Institute of Higher Education and Research, Chennai, IND; 5 Radiology, Sri Ramachandra Institute of Higher Education and Research, Chennai, IND

**Keywords:** candida tropicalis, febrile neutropenia, fluconazole susceptibility, galactomannan, india, invasive aspergillosis, invasive fungal infection, mucormycosis, paediatric haemato-oncology

## Abstract

Background: Invasive fungal infections (IFIs) are a leading cause of morbidity and mortality in paediatric haemato-oncology (PHO) patients. Empirical antifungal therapy in high-risk children must be informed by local epidemiological, species-level and antifungal-susceptibility data, yet contemporary Indian paediatric cohort data remain limited.

Objective: To describe the clinical, microbiological, therapeutic and outcome profile of probable and proven IFI in a tertiary care South Indian PHO unit over five years, and to benchmark the findings against contemporary international literature.

Methods: Retrospective observational cohort study of children aged ≤18 years admitted with probable or proven IFI (EORTC/MSGERC 2020 criteria) between January 2019 and December 2024. Baseline demographics, underlying disease, neutropenia profile, clinical presentation, mycological workup (cultures with CLSI M27 susceptibility testing, serum galactomannan (GM), beta-D-glucan and bronchoalveolar lavage), imaging, antifungal prophylaxis and therapy, central venous access device (CVAD) management and infection-episode outcomes were extracted and analysed descriptively.

Results: Among 580 febrile PHO admissions, 33 episodes of probable/proven IFI occurred in 23 patients (overall incidence 5.8%). Median age was 8.5 years (range 1.5-17); 12/23 (52.2%) were male. Haemato-lymphoid malignancy was the underlying diagnosis in 17/23 (73.9%); two children had inborn errors of immunity (autoimmune lymphoproliferative syndrome, n = 1; hyper-IgM syndrome, n = 1). Three patients (13.0%) were haploidentical haematopoietic stem cell transplantation recipients. Neutropenia (ANC < 1500 cells/mm³) was present in 19/23 (82.6%), and profound neutropenia (ANC < 100 cells/mm³) in 7/23 (30%) at IFI onset. Ten patients (43.5%) had a breakthrough infection on prophylaxis. *Candida* spp. and *Aspergillus* spp. were the two dominant pathogen groups (each 43.5%; n = 10), followed by *Mucorales* (8.7%; n = 2) and *Geotrichum* (*Magnusiomyces*) capitatum (4.3%; n = 1). All 20 *Candida* episodes presented as fungemia; *Candida tropicalis* was the single most common isolate (12/20; 60.0%), and 20/20 (100%) were fluconazole-susceptible on CLSI M27 testing. Fluconazole was the first-line therapeutic agent in candidemia, informed by universal in vitro susceptibility and CVAD was removed in all 10 *Candida*-infected patients (100%). Serum GM was positive in 8/23 (34.8%). Radiological fungal pneumonia was documented in 10/23 (43.5%), with air-crescent, ground-glass, nodular and consolidative patterns in 11%, 10%, 7% and 15% of the cohort, respectively; sinusitis was identified in 2/23 (8.6%). Crude all-cause mortality was 6/23 (26.1%): 0 in the *Candida* group, 3/10 (30%) in aspergillosis, 2/2 (100%) in mucormycosis and 1/1 (100%) in *Geotrichum* infection.

Conclusion: In this contemporary South Indian PHO cohort, IFI incidence was 5.8% with a *Candida*-*Aspergillus* co-dominance, a marked *C. tropicalis* preponderance, universal fluconazole susceptibility permitting fluconazole-based first-line candidemia therapy, no candidaemia-attributable deaths alongside 100% CVAD removal, and high mortality in the small numbers of patients with mould infection. These data support locally informed empirical antifungal selection, aggressive CVAD-based source control and structured deployment of imaging and biomarker-driven diagnosis of invasive mould disease in Indian PHO.

## Introduction

Invasive fungal infection (IFI) remains one of the complications we most fear in children receiving intensive treatment for malignancy. Prolonged and profound neutropenia, cytotoxic mucosal injury, indwelling central venous access, broad-spectrum antibacterial exposure and, in a growing minority of children, allogeneic haematopoietic stem cell transplantation (HSCT) together predispose paediatric haemato-oncology (PHO) patients to invasive yeast and mould disease. Invasive fungal disease has historically accounted for a disproportionate share of treatment-related mortality in childhood acute myeloid leukaemia (AML), high-risk acute lymphoblastic leukaemia (ALL) and post-transplant populations, with attributable case fatality varying widely between geographies and between pathogen groups [1].

The revised 2020 European Organization for Research and Treatment of Cancer and Mycoses Study Group Education and Research Consortium (EORTC/MSGERC) consensus definitions brought greater diagnostic granularity, re-classifying host factors, integrating serum beta-D-glucan (BDG) and galactomannan (GM) thresholds, and formally recognising Mucorales-specific criteria [2]. The 8th European Conference on Infections in Leukaemia (ECIL-8) paediatric guidelines then refined recommendations on mould-active prophylaxis, empirical antifungal therapy and species-directed treatment in children with cancer or after transplantation [3]. The Children's Oncology Group and international expert panel guideline of Lehrnbecher et al. addresses systemic antifungal prophylaxis in paediatric cancer and HSCT recipients specifically [4], and the European Society of Clinical Microbiology and Infectious Diseases and European Confederation of Medical Mycology (ESCMID-ECMM) guideline of Warris et al. further refines the diagnosis and management of invasive aspergillosis in neonates and children [5].

Contemporary global series report cumulative IFI incidences of 5-20% in paediatric acute leukaemia cohorts, with *Candida* spp. and *Aspergillus* spp. accounting for most confirmed disease. Kazakou et al., in a 16-year Greek paediatric haemato-oncology cohort of 297 patients, reported IFI rates of 10.5% in ALL, 18.2% in AML and 2.8% in non-Hodgkin lymphoma [6]. The prospective All India Institute of Medical Sciences (AIIMS), Delhi, series of Kumar et al. reported a substantially higher prevalence of 22.97%, although that figure applies to the persistent-febrile-neutropenia subset of paediatric acute leukaemia rather than to an unselected cohort [7].

Indian mould epidemiology has a distinct signature. The prospective multicentre Indian study by Prakash et al. characterised mucormycosis epidemiology, diagnosis and treatment across four tertiary centres [8], and Skiada et al. reviewed the contemporary international epidemiology and diagnosis of the same disease [9]. Prakash and Chakrabarti put the estimated Indian prevalence of mucormycosis at roughly 70 times that in global data [10].

For candidemia, the five-year AIIMS Delhi surveillance by Xess et al. documented *C. tropicalis* as the leading species in North Indian tertiary practice [11]. Kothari and Sagar confirmed the predominance of non-albicans *Candida* at a separate Indian tertiary centre [12]. Dewan et al. looked specifically at candidemia in patients with haematological malignancy at an Indian tertiary-care centre and found *C. tropicalis* the commonest isolate at 46.7%, with non-albicans species outnumbering *C. albicans* by 2.75-1% [13].

Rare yeasts have become clinically consequential in the profoundly neutropenic host. The Italian multicentre retrospective series of Girmenia et al. documented *Trichosporon* and *Geotrichum capitatum* infection in patients with haematological malignancy, with high mortality [14]. Schuermans et al. reported breakthrough *Saprochaete capitata* infection in patients already receiving echinocandins, which characterised this organism further [15].

Non-culture diagnostics matter for invasive mould disease. Zhou et al. quantified the diagnostic value of serum and bronchoalveolar lavage (BAL) GM testing in non-neutropenic invasive pulmonary aspergillosis and found lavage GM markedly more sensitive than serum GM, 64.9% against 24.3% at an index of 1.0 [16]. Salvatore et al. established BDG in cerebrospinal fluid as a biomarker for *Candida* and *Aspergillus* infection of the paediatric central nervous system [17]. Badiee et al. evaluated nested polymerase chain reaction, GM enzyme immunoassay and BDG together in paediatric invasive aspergillosis [18].

Directed BAL adds complementary yield in paediatric oncology. Efrati et al. documented the utility of fiberoptic bronchoscopy with BAL in children with primary immunodeficiency and cancer [19]. Nadimpalli et al. reported the diagnostic yield of BAL in immunocompromised children with malignant and non-malignant disorders, isolating a potential pathogen in 31% of subjects [20].

Against that background, contemporary Indian data covering the full pathogen, susceptibility and outcome spectrum of paediatric IFI within a single institutional cohort, taking in *Candida*, *Aspergillus*, Mucorales and the rare yeasts together, remain scarce. We report a five-year retrospective experience from a tertiary-care PHO unit in South India. Our primary objective was to describe the clinical, microbiological, susceptibility and outcome profile of probable and proven IFI in 23 children with 33 infection episodes. Our secondary objective was to place those descriptive findings beside the international and Indian literature.

## Materials and methods

Study design and setting

This retrospective observational cohort study was conducted in the Department of Paediatric Haematology-Oncology at Sri Ramachandra Institute of Higher Education and Research (SRIHER), a tertiary-care university hospital in Chennai, India. We screened consecutive paediatric haemato-oncology admissions between 1 January 2019 and 31 December 2024. The Institutional Ethics Committee of SRIHER approved the study (approval number CSP-MED/26/JAN/124/20, dated 29 January 2026). Individual informed consent was waived because the design was retrospective and the data anonymised.

Patient selection and case definitions

We screened the case records of all children aged 18 years or younger admitted with febrile illness during the study period, working from the medical records department and the electronic medical record of the institute. Patients were included if they met the revised 2020 EORTC/MSGERC consensus criteria for proven or probable invasive fungal disease [2]. Possible IFI was recorded for descriptive completeness, in line with reporting practice in the ECIL-8 paediatric framework [3]. Proven disease required histopathological, microscopic or sterile-site culture demonstration of the pathogen. Probable disease required a host factor, a clinical feature and mycological evidence, whether by culture or by biomarker positivity, to occur together. Patients whose records were too incomplete to permit classification were excluded. Two of the treating paediatric haemato-oncologists classified every case independently, working from the case record, the mycology report and the reported imaging, and disagreements were settled by consensus with the microbiologist and radiologist co-authors. We defined a recurrent episode as a new microbiologically or radiologically documented invasive fungal episode arising after the preceding episode had documented resolution or an episode caused by a different pathogen. Episodes less than 14 days apart with no documented interval of resolution were counted as one episode. Where a required variable was missing from the record, we reported it as not available and reduced the denominator for that variable accordingly, and we imputed no values. Two thresholds for neutropenia appear in this paper, and they do different work. For EORTC/MSGERC host-factor classification, the qualifying criterion was severe neutropenia, an absolute neutrophil count (ANC) below 500 cells/mm³ documented for more than 10 days. The wider ANC threshold of 1,500 cells/mm³ appears only in the descriptive cohort tables, where it characterises the depth of cytopenia at presentation, and it was never used to establish host-factor eligibility.

Data collection

A structured proforma was used to abstract the following. Demographics, the underlying haematological, oncological or immunodeficiency diagnosis, disease phase and chemotherapy intensity at IFI onset, history and type of HSCT, use and agent of antifungal prophylaxis, clinical presentation, duration and depth of neutropenia, haematological, hepatic and renal laboratory parameters, presence and management of central venous access devices (CVAD), serum GM and BDG results, imaging findings on chest and sinus computed tomography (CT), BAL results where performed, antifungal therapy given, and the outcome of the infectious episode. Neither cross-sectional imaging nor BAL was protocolised. A chest CT was obtained where fever persisted beyond 96 hours despite broad-spectrum antibacterial therapy or where respiratory signs or an abnormal chest radiograph were present. Sinus CT was obtained where sino-orbital symptoms or signs were present. Bronchoscopy with BAL was undertaken only where a pulmonary infiltrate was present, a non-invasive diagnosis had not been reached and the treating team judged the child fit for the procedure.

Institutional antifungal prophylaxis protocol

Antifungal prophylaxis at the study centre was risk-stratified by underlying disease and clinical phase, in line with the ECIL-8 paediatric recommendations [3] and the Lehrnbecher et al. prophylaxis guideline [4]. Patients with relapsed ALL and patients with AML received voriconazole, and those with AML who developed severe neutropenia or mucositis received micafungin instead. Patients after HSCT received micafungin until engraftment, then voriconazole or posaconazole, continued to day 100 after transplantation. Children with solid tumours and profound post-chemotherapy neutropenia were started on fluconazole until neutrophil recovery. Children on upfront ALL or high-grade lymphoma therapy received no systemic antifungal prophylaxis. Where an azole was given alongside vincristine, the vincristine dose was halved to manage the known interaction. Therapeutic drug monitoring of mould-active azoles was not available to us during the study period. We defined breakthrough infection as any proven or probable IFI arising while a mould-active or yeast-active prophylactic agent was being given. Chemotherapy comprised an augmented Berlin-Frankfurt-Munster (BFM) protocol for ALL, a Children's Oncology Group protocol for AML and a myeloablative conditioning regimen containing busulfan, fludarabine or total body irradiation before transplantation.

Microbiological methods

Blood cultures were processed in an automated continuous-monitoring system (BacT/ALERT 3D, bioMerieux, Marcy-l'Étoile, France). The mean turnaround time for fungal blood cultures was 72 hours. *Candida* isolates were identified to species level on chromogenic agar and confirmed by matrix-assisted laser desorption ionisation time-of-flight (MALDI-TOF) mass spectrometry, and every isolate underwent in vitro susceptibility testing to fluconazole, voriconazole and the echinocandins by CLSI M27 methodology. Serum GM was measured with the Platelia Aspergillus enzyme immunoassay (Bio-Rad Laboratories Inc., Hercules, California) and reported as an optical density index, an index of 1.0 or greater on a single serum sample being taken as positive in line with the EORTC/MSGERC 2020 criteria [2]. Susceptibility was interpreted using the CLSI broth microdilution reference method with species-specific clinical breakpoints for fluconazole and reported categorically as susceptible, susceptible-dose-dependent or resistant. We did not use numerical minimum inhibitory concentration values, including MIC50 and MIC90, in this analysis. BDG was measured by the Fungitell assay with a cut-off of 80 pg/mL or greater. Sinus and lung biopsy specimens underwent potassium hydroxide (KOH) microscopy, fungal culture and histopathological examination.

Definitions of outcome

The primary outcome was infection-attributable case fatality by causative pathogen group at the end of the infection episode, taken as discharge or death. We defined an attributable death as one occurring during an active, microbiologically or radiologically documented invasive fungal episode with no alternative proximate cause. Secondary outcomes were the proportion of episodes classified as breakthrough infection on prophylaxis, CVAD removal in candidemia and the diagnostic yield of serum GM, BDG and BAL.

Statistical analysis

Categorical variables were summarised as absolute frequencies and proportions. Continuous variables were summarised as median with range or as mean with standard deviation, according to distribution assessed by the Shapiro-Wilk test. Between-group comparisons used the Fisher exact test for categorical outcomes, the Mann-Whitney U test for non-parametric continuous variables and the Student t-test for normally distributed continuous variables, with the test statistic reported in each case. Where differences in proportions across three or more categories were assessed, we used the chi-square test, and analysis of variance was reserved for parametric multi-group continuous comparisons. In line with journal policy, wherever a p-value is reported in the text or in a table legend, the corresponding test statistic and the name of the test are given alongside it. A two-sided p-value below 0.05 was pre-specified as statistically significant. Given the descriptive intent and the modest sample size, all inferential p-values should be read as exploratory. Pathogen-specific mortality involves very small numerators, so it is summarised as proportions with Wilson score 95% confidence intervals (CI) rather than as inferential p-values. Analyses were performed in IBM SPSS Statistics for Windows, Version 26 (Released 2019; IBM Corp., Armonk, New York).

## Results

Cohort characterisation and study flow

We screened 580 febrile admissions over the five-year study period and identified 33 episodes of probable or proven IFI in 23 unique patients. The patient-level cumulative incidence was 5.8% (23 of 396 unique febrile paediatric haemato-oncology patients screened). At the episode level, there were 33 episodes among 580 febrile admissions. Patients are the primary unit of analysis throughout this paper. Concurrent bacterial blood cultures were sterile in every patient with proven or probable invasive fungal disease, and no patient had concurrent COVID-19 illness. Study flow, pathogen partitioning and pathogen-specific mortality are shown in Figure 1.

**Figure 1:**
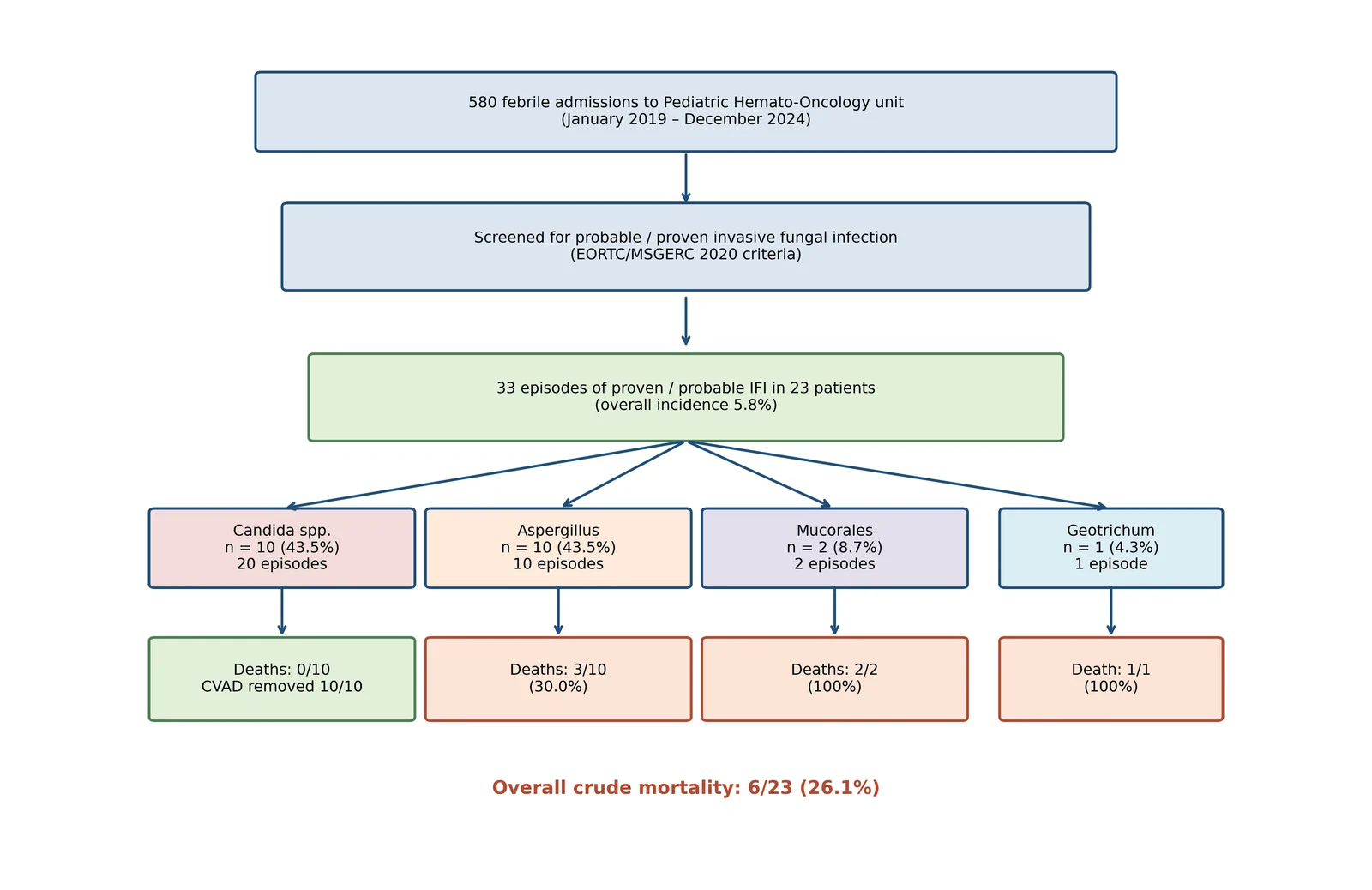
Study flow diagram showing case identification, causative pathogen distribution and pathogen-specific mortality. A total of 33 IFI episodes occurred in 23 paediatric haemato-oncology patients from January 2019 to December 2024. Pathogen-specific mortality proportions shown in this flow diagram are reported as proportions with Wilson 95% CI rather than as an inferential p-value, because the pathogen-specific numerators are very small. The values are *Candida,* 0 of 10 (95% CI 0.0 to 27.8); *Aspergillus*, 3 of 10 (95% CI 10.8 to 60.3); Mucorales, 2 of 2 (95% CI 34.2 to 100.0); and *Geotrichum*, 1 of 1 (95% CI 20.7 to 100.0).

Demographic, clinical and haematological characteristics

Median age at IFI onset was 8.5 years (range 1.5-17 years), and 12 of 23 patients (52.2%) were male. Haemato-lymphoid malignancy was the dominant substrate, occurring in 17 of 23 patients (73.9%), comprising acute lymphoblastic leukaemia in 10 patients, three of whom had relapsed disease, acute myeloid leukaemia in three patients, and lymphoma in four patients. Solid tumours accounted for three of 23 patients (13.0%), two neuroblastomas and one medulloblastoma. Two children (8.7%) had an inborn error of immunity: one had autoimmune lymphoproliferative syndrome (ALPS) and one had hyper-IgM syndrome. One child (4.3%) had transfusion-dependent thalassaemia. Three patients (13.0%) had undergone prior haploidentical HSCT for relapsed ALL, refractory AML and refractory Hodgkin lymphoma, respectively, and no matched-sibling or matched-unrelated-donor grafts were represented in the IFI cohort. Ten patients (43.5%) were receiving systemic antifungal prophylaxis when IFI was diagnosed and therefore met our definition of breakthrough infection.

Cytopenias were near-universal at IFI onset. We recorded anaemia in 20 of 23 patients (85%), thrombocytopenia in 16 of 23 (70%) and neutropenia, an ANC below 1500 cells/mm³, in 19 of 23 (82.6%); of these, 7 of 23 patients (30%) had profound neutropenia, with an ANC below 100 cells/mm³. Hepatic transaminase or bilirubin derangement was documented in 7 of 23 (30%) and renal derangement in 1 of 23 (4.3%). Fever was present in every patient. Respiratory symptoms occurred in 15 of 23 (65.2%), and gastrointestinal symptoms in 5 of 23 (21.7%). Baseline demographic, haematological and biochemical characteristics are summarised in Table 1.

**Table 1:** Baseline demographic, clinical, haematological and biochemical characteristics of the study cohort (n = 23). Breakthrough infection is defined per the EORTC/MSGERC 2020 criteria [2]. Statistical test note. This is a descriptive summary of a single cohort of 23 patients. No between-group inferential comparison is performed within this table, so no test statistic or p-value is reported. Where subgroup comparisons were performed elsewhere in the manuscript, the Fisher exact test was applied for categorical variables and the Mann-Whitney U test for non-parametric continuous variables, with the test statistic reported. SD: Standard deviation; ANC: Absolute neutrophil count; HSCT: Haematopoietic stem cell transplantation; ALPS: Autoimmune lymphoproliferative syndrome; CVAD: Central venous access device; IFI: Invasive fungal infection.

Variable	n/value	%
Age at IFI onset (years), median (range)	8.5 (1.5-17)	-
Sex (Male)	12	52.2
Sex (Female)	11	47.8
Duration of illness at presentation (days), mean ± SD	14 ± 3	-
Underlying diagnosis: Haemato-lymphoid malignancy	17	73.9
Underlying diagnosis: Solid tumour	3	13.0
Underlying diagnosis: Inborn error of immunity (ALPS n = 1, hyper-IgM n = 1)	2	8.7
Underlying diagnosis: Transfusion-dependent thalassaemia	1	4.3
Prior haploidentical HSCT	3	13.0
Systemic antifungal prophylaxis at IFI onset (breakthrough per EORTC/MSGERC 2020 criteria [2])	10	43.5
Anaemia at IFI onset	20	85.0
Thrombocytopenia at IFI onset	16	70.0
Neutropenia (ANC <1500 cells/mm³)	19	82.6
Profound neutropenia (ANC <100 cells/mm³)	7	30.0
Hepatic transaminase/bilirubin derangement	7	30.0
Renal parameter derangement	1	4.3
Presenting symptom: Fever	23	100.0
Presenting symptom: Respiratory (cough, tachypnoea and hypoxia)	15	65.2
Presenting symptom: Gastrointestinal (abdominal pain and diarrhoea)	5	21.7
CVAD in situ at IFI onset	15	65.2

Distribution of causative fungal pathogens

*Candida* spp. and *Aspergillus* spp. were the two dominant pathogen groups, each accounting for 10 patients (43.5%). Mucorales caused two patient-level infections (8.7%), and *Geotrichum* (Magnusiomyces) *capitatum* a single episode (4.3%). The 10 patients with *Candida* infection contributed 20 fungaemic episodes, whereas *Aspergillus*, Mucorales and *Geotrichum* each contributed one episode per affected patient. The overall pathogen distribution is shown in Figure 2.

**Figure 2:**
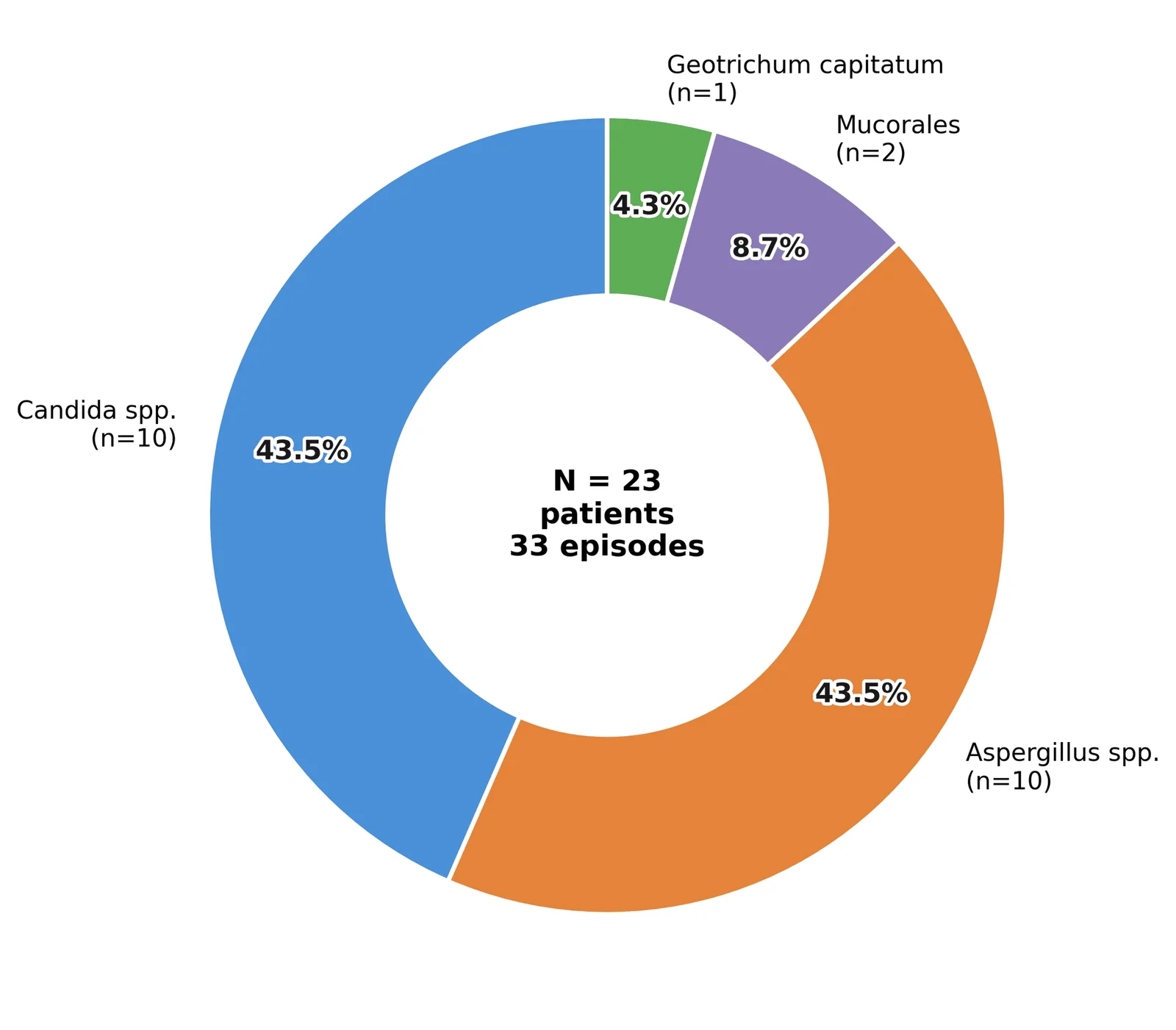
Distribution of causative fungal pathogen groups across 23 unique paediatric patients with probable or proven invasive fungal disease. *Candida* spp. and *Aspergillus* spp. co-dominate and together account for 87% of the cohort. Statistical test note. This figure displays the descriptive distribution of pathogen groups across a single cohort of 23 patients and does not depict a between-group inferential comparison, so no test statistic or p-value is attached to it.

*Candida* species distribution and susceptibility

All 20 *Candida* episodes presented as fungemia, and non-albicans species accounted for 17 of 20 isolates (85.0%). *C. tropicalis* was the single most common species, accounting for 12 of 20 isolates (60.0%), followed by *C. parapsilosis* in 4 of 20 (20.0%), *C. albicans* in 3 of 20 (15.0%) and *C. guilliermondii* in 1 of 20 (5.0%). In vitro susceptibility testing by CLSI M27 showed fluconazole susceptibility in all 20 isolates (100%), with no dose-dependent susceptible or resistant strains identified. This universal susceptibility informed our first-line therapeutic choice. We compare the species distribution with published Indian series in the 'Discussion' section.

Microbiological and imaging findings

Serum GM, measured using the Platelia Aspergillus enzyme immunoassay (Bio-Rad Laboratories Inc., Hercules, California) at an index of 1.0 or greater, was positive in 8 of 23 tested patients (34.8%). GM was requested where invasive mould disease was clinically or radiologically suspected, so this proportion is the diagnostic yield within a selected subgroup rather than a population positivity rate, and it is not a measure of assay sensitivity. For reference, Badiee et al. reported a serum GM sensitivity of 90% and specificity of 92% in a paediatric haematology-oncology cohort [18], while Zhou et al. found serum GM far less sensitive than lavage GM in non-neutropenic invasive pulmonary aspergillosis [16]. BDG testing was performed in only three patients and was positive in two of three (66.7%). That denominator is far too small to support inference, and the published paediatric experience is itself mixed. Salvatore et al. detected cerebrospinal-fluid BDG in all nine children with proven or probable *Candida* or *Aspergillus* central nervous system infection [17], whereas Badiee et al. reported a serum BDG sensitivity of only 50% in children at risk of invasive aspergillosis [18]. BAL was performed in two patients with pulmonary infiltrates and yielded a fungal diagnosis in one of two (50%), congruent with the yield in immunocompromised children reported by Efrati et al., who recovered a definite organism in 53.2% of procedures [19], and by Nadimpalli et al., who isolated a potential pathogen in 31% [20]. Cross-sectional imaging identified invasive fungal pneumonia in 10 of 23 (43.5%) and radiological sinusitis in 2 of 23 (8.6%). Among the patients with disseminated disease, one with pulmonary aspergillosis had spread to the maxillary sinus and one with mucormycosis had spread to the right orbit and frontal sinus. Among patients with fungal pneumonia, the predominant CT patterns, expressed as a proportion of the whole cohort of 23, were consolidation in 15%, the air-crescent sign in 11%, ground-glass opacity in 10% and nodular lesions in 7%. Microbiological findings and diagnostic-test performance are summarised in Table 2, and imaging patterns by pathogen group and CT pattern are summarised in Table 3.

**Table 2:** Microbiological profile of invasive fungal isolates, antifungal susceptibility and diagnostic-test performance in the present cohort. The EORTC/MSGERC 2020 categorisation is applied throughout [2]. Non-albicans *Candida* accounted for 85% (17 of 20) of candidaemic episodes, and CLSI M27 susceptibility testing was performed on every *Candida* isolate. Statistical test note. This table reports descriptive microbiological and diagnostic-test proportions in a single cohort of 23 patients. No between-group inferential comparison is performed within it, so no test statistic or p-value is reported. GM: Galactomannan; BAL: Bronchoalveolar lavage; BDG: Beta-D-glucan.

Microbiological parameter	n / N	%
*Candida* spp., patients	10/23	43.5
*C. tropicalis* (episodes)	12/20	60.0
*C. parapsilosis* (episodes)	4/20	20.0
*C. albicans* (episodes)	3/20	15.0
*C. guilliermondii* (episodes)	1/20	5.0
All *Candida* episodes fluconazole-susceptible (CLSI M27)	20/20	100.0
Echinocandin-resistant *Candida*	0/20	0.0
*Aspergillus* spp., patients	10/23	43.5
Probable invasive aspergillosis	4/10	40.0
Possible invasive aspergillosis	6/10	60.0
Mucorales, patients	2/23	8.7
*Geotrichum* (Magnusiomyces) capitatum, patients	1/23	4.3
Serum GM positive (index ≥ 1.0)	8/23	34.8
Serum BDG performed	3/23	13.0
BDG positive (≥80 pg/mL)	2/3	66.7
BAL performed	2/23	8.7
BAL fungal-positive	1/2	50.0

**Table 3:** Site of infection, computed tomography (CT) imaging patterns and imaging findings by pathogen group. Numbers represent patients or, for CT sub-patterns, the proportion of the whole cohort of 23 showing each predominant radiological finding. Statistical test note. Comparison of pulmonary-infiltrate distribution across the four pathogen groups used the Fisher exact test, with a test statistic of 22.7 and p < 0.001. Comparison of sinusitis distribution used the Fisher exact test, with a test statistic of 11.3 and p = 0.008. Where an expected cell count was below 5, the Fisher exact test was used in preference to the Pearson chi-square test. CT: Computed tomography.

Parameter	*Candida* (n = 10) n (%)	*Aspergillus* (n = 10) n (%)	Mucorales (n = 2) n (%)	*Geotrichum* (n = 1) n (%)	Total (n = 23) n (%)
Bloodstream infection (fungemia)	10 (100%)	0 (0%)	0 (0%)	1 (100%)	11 (47.8%)
Pulmonary infiltrate on CT	0 (0%)	9 (90%)	1 (50%)	0 (0%)	10 (43.5%)
Consolidation pattern	Cohort-wide 15% (n = 23)				
Air-crescent sign	Cohort-wide 11% (n = 23)				
Ground-glass opacity	Cohort-wide 10% (n = 23)				
Nodular lesions	Cohort-wide 7% (n = 23)				
Sinusitis on CT	0 (0%)	0 (0%)	2 (100%)	0 (0%)	2 (8.6%)
Disseminated/multifocal disease	0 (0%)	1 (10%)	1 (50%)	0 (0%)	2 (8.7%)

Antifungal prophylaxis, breakthrough profile and first-line therapy

Our prophylaxis protocol stratified regimens by risk. Patients with relapsed ALL or AML received voriconazole, and those with AML complicated by severe neutropenia or mucositis received micafungin. Patients after HSCT received micafungin until engraftment and then voriconazole or posaconazole, and children with solid tumours and profound post-chemotherapy neutropenia received fluconazole. Ten of 23 patients (43.5%) developed IFI while on this stratified prophylaxis and were classified as breakthrough infections. That proportion is congruent with the residual risk of IFI during prophylaxis recognised in ECIL-8 [3] and by Lehrnbecher et al. [4], and it can be considered alongside the randomised comparison of posaconazole with fluconazole or itraconazole prophylaxis in AML and myelodysplastic syndrome reported by Cornely et al. [21].

Our first-line antifungal therapy in candidemia was fluconazole, a practice grounded in the fluconazole susceptibility of all 20 *Candida* isolates (100%) according to CLSI M27 testing. That differs from the Infectious Diseases Society of America (IDSA) candidiasis guideline by Pappas et al., which specifies an echinocandin as the default first-line agent for candidemia [22]. We present our practice as a locally justified, susceptibility-guided divergence and not as a universal recommendation. Liposomal amphotericin B was the preferred first-line agent in all mucormycosis and *Geotrichum* episodes, consistent with the ESCMID-ECMM paediatric aspergillosis guidance [5] and the IDSA aspergillosis guideline by Patterson et al. [23]. Voriconazole was used first-line in probable invasive aspergillosis where clinically appropriate, consistent with the randomised controlled trial by Herbrecht et al., which established a 12-week survival benefit of voriconazole over deoxycholate amphotericin B [24], and with the open-label paediatric voriconazole experience of Walsh et al. [25]. A CVAD was in situ in 15 of 23 patients (65.2%) at IFI onset, comprising a peripherally inserted central catheter in 6, a chemoport in 5 and a non-tunnelled central venous catheter in 4, and it was removed as part of source control in all 10 patients with candidemia (100%). In the patients with invasive aspergillosis the infection was pulmonary without bloodstream involvement, so the device was not the source and was not removed. Zaoutis et al. identified candidemia persisting beyond three days with a central venous catheter left in situ as an independent risk factor for disseminated paediatric candidiasis, with an odds ratio of 3.0 (95% CI 1.2 to 7.8) [26]. Karadag-Oncel et al. reinforced the importance of prompt catheter removal, reporting a 20.5-fold to 23-fold increase in 30-day mortality when the catheter was not removed [27]. Fisher et al. showed in a retrospective cohort of 285 children that catheter retention was independently associated with death, with an odds ratio of 2.50 (95% CI 1.06 to 5.91) [28]. Treatment allocation and device management by pathogen group are summarised in Table 4.

**Table 4:** Antifungal prophylaxis, therapy allocation, central venous access device (CVAD) management and outcome by pathogen group. Prophylaxis proportions reflect the institutional risk-stratified protocol. First-line therapy percentages are row-wise relative to the pathogen-group denominator. Fluconazole first-line for candidemia was supported by fluconazole susceptibility in all isolates on CLSI M27 testing. Statistical note. Pathogen-specific mortality is reported as proportions with Wilson 95% CI rather than as an inferential p-value, given the very small numerators. The values are *Candida,* 0 of 10, 0.0% (95% CI 0.0 to 27.8); *Aspergillus*, 3 of 10, 30.0% (95% CI 10.8 to 60.3); Mucorales 2 of 2, 100% (95% CI 34.2 to 100.0) and *Geotrichum,* 1 of 1, 100% (95% CI 20.7 to 100.0). Comparison of first-line antifungal agents across pathogen groups used the Fisher exact test, with a test statistic of 44.2 and p < 0.001. Comparison of device removal used the Fisher exact test, with a test statistic of 16.9 and p = 0.001. CVAD: Central venous access device. ALL: Acute lymphoblastic leukaemia; AML: Acute myeloid leukaemia; HSCT: Haematopoietic stem cell transplantation; IFI: Invasive fungal infection.

Parameter	*Candida* (n = 10) n (%)	*Aspergillus* (n = 10) n (%)	Mucorales (n = 2) n (%)	*Geotrichum* (n = 1) n (%)	Total (n = 23) n (%)
First-line therapy, fluconazole	10 (100%)	0 (0%)	0 (0%)	0 (0%)	10 (43.5%)
First-line therapy, voriconazole	0 (0%)	6 (60%)	0 (0%)	0 (0%)	6 (26.1%)
First-line therapy, liposomal amphotericin B	0 (0%)	4 (40%)	2 (100%)	1 (100%)	7 (30.4%)
CVAD in situ at IFI onset	10 (100%)	3 (30%)	1 (50%)	1 (100%)	15 (65.2%)
CVAD removed as source control	10 (100%)	0 (0%)	0 (0%)	0 (0%)	10 (43.5%)
Deaths (episode-level)	0 (0%)	3 (30%)	2 (100%)	1 (100%)	6 (26.1%)

Outcomes and mortality

Crude in-hospital all-cause mortality was 6 of 23 (26.1%, Wilson 95% CI 12.5 to 46.5), and it varied by pathogen. There were no deaths in the *Candida* group, 0 of 10, 0.0% (95% CI 0.0 to 27.8). Three of 10 patients with invasive aspergillosis died (30.0%, 95% CI 10.8 to 60.3), all of them receiving intensive myelosuppressive chemotherapy at the time. Both patients with mucormycosis died (2 of 2, 100%; 95% CI 34.2 to 100.0), as did the single patient with *Geotrichum capitatum* infection, 1 of 1, 100% (95% CI 20.7 to 100.0), who was debilitated and on prolonged broad-spectrum antibacterial therapy. Because these pathogen-specific numerators are very small, mortality is summarised as proportions with Wilson score 95% CIs rather than as inferential p-values. Pathogen-specific mortality is shown in Figure 3.

**Figure 3:**
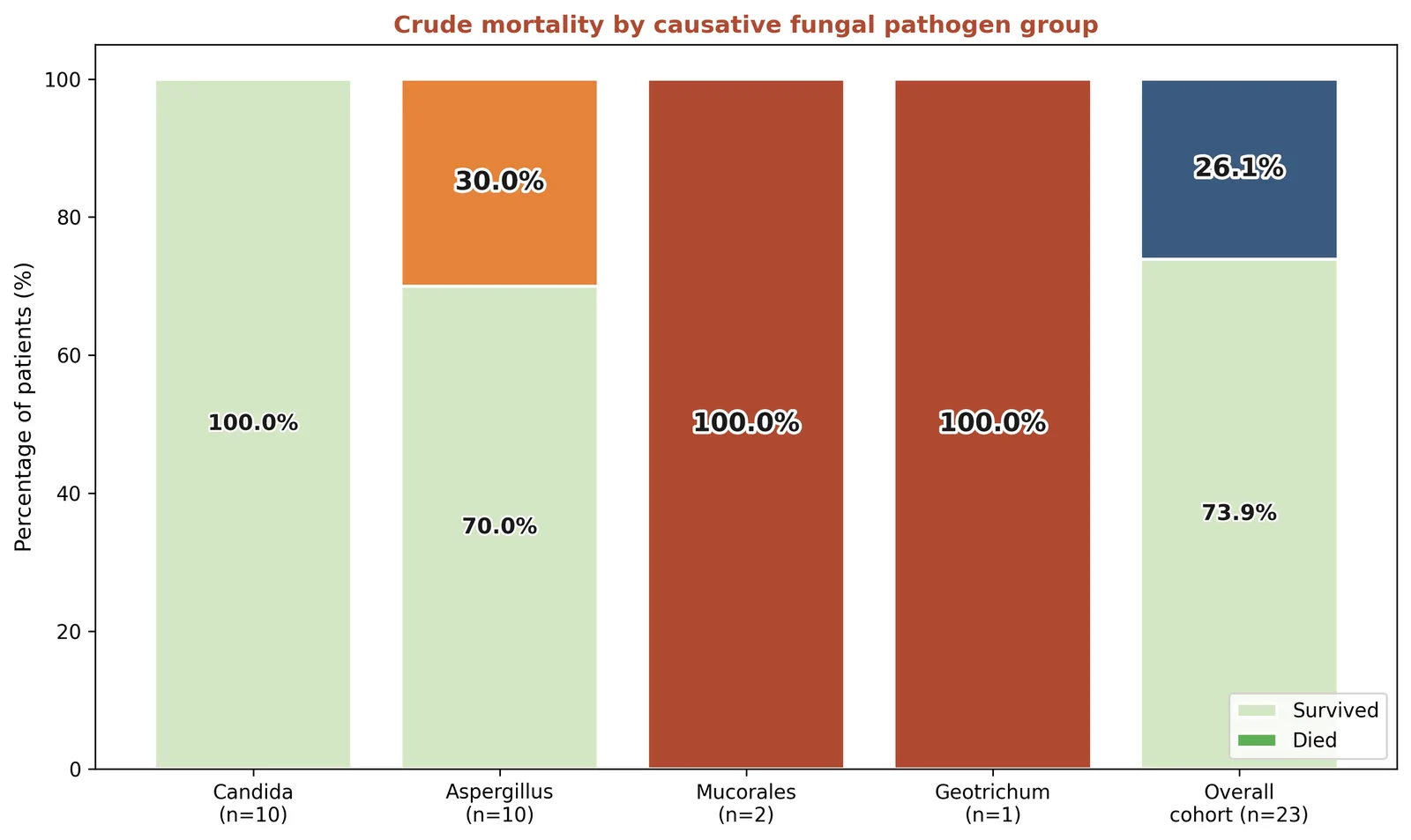
Crude in-hospital mortality by causative fungal pathogen group. Mould infections, meaning invasive aspergillosis and mucormycosis, together with the single *Geotrichum* episode, accounted for all six deaths in the cohort, and no candidemia-attributable death was recorded. Statistical test note. The pathogen-specific crude mortality proportions displayed here, *Candida,* 0 of 10 at 0.0%; *Aspergillus,* 3 of 10 at 30.0%; Mucorales, 2 of 2 at 100%; and *Geotrichum,* 1 of 1 at 100%, are reported as proportions with Wilson 95% CIs rather than as an inferential p-value, in view of the very small numerators.

## Discussion

This five-year single-centre South Indian cohort captures the contemporary spectrum of invasive fungal disease in a real-world, resource-adequate tertiary setting. Four findings stand out. *Candida* and *Aspergillus* co-dominated, with a marked *C. tropicalis* preponderance among candidaemic isolates. Every *Candida* isolate was fluconazole-susceptible, which supported a locally informed fluconazole-based first-line therapy. There were no candidemia-attributable deaths, in contrast to death in both patients with mucormycosis and in the single patient with *Geotrichum* infection. The cumulative incidence of proven or probable IFI among febrile PHO patients was 5.8%. Taken together, these findings re-affirm prevailing epidemiological expectations and, in specific respects, refine them.

Incidence compared with global and Indian cohorts

Our 5.8% incidence sits at the lower end of the reported global range for paediatric haemato-oncology IFI. Kazakou et al., in a 16-year Greek cohort of 297 patients, reported rates of 10.5% in ALL, 18.2% in AML and 2.8% in non-Hodgkin lymphoma, with *Candida* and *Aspergillus* predominant [6]. The AIIMS Delhi prospective series by Kumar et al. reported a substantially higher prevalence of 22.97%, but that figure comes from the persistent febrile neutropenia subset of acute leukaemia, a denominator enriched for high-risk children [7]. Our denominator was all febrile PHO patients, which explains the lower apparent incidence. The comparison is a reminder that an incidence figure means little without its reference population. Ten of 23 patients (43.5%) were on antifungal prophylaxis at IFI onset, so nearly half our episodes were breakthrough infections. That proportion is congruent with the residual risk of IFI despite mould-active prophylaxis established by ECIL-8 [3], by Lehrnbecher et al. [4] and Cornely et al. [21].

*Candida* species distribution and the fluconazole-susceptibility signal

The most striking microbiological finding was the dominance of *C. tropicalis*, which accounted for 60.0% of candidaemic episodes (12 of 20), followed by *C. parapsilosis* at 20.0% and *C. albicans* at 15.0%. That pattern is consistent with the two established Indian tertiary-centre series and lies at the higher end of them, with Xess et al. at AIIMS Delhi reporting 35.3% [11] and Dewan et al. in a haematological malignancy cohort reporting 46.7% [13]. The predominance of non-albicans species, which accounted for 85% of our candidaemic episodes, fits the broader Indian literature, including the AIIMS cohort of Xess et al. [11], the tertiary-centre series of Kothari and Sagar [12] and the series of Dewan et al. [13], all of which document a pattern of nosocomial candidemia distinct from Western cohorts, where *C. albicans* has historically predominated. Species-directed susceptibility testing and evaluation of host immunity matter particularly in children with atypical non-albicans *Candida* infection, as illustrated by a reported *C. parapsilosis* infection in an HIV-negative infant with profound CD4+ lymphopenia [29].

Equally consequential is the fluconazole susceptibility of all 20 *Candida* isolates in vitro. That profile, obtained systematically using CLSI M27 methodology, supported our choice of fluconazole as first-line therapy in candidemia. The practice diverges from the IDSA and ECIL default of an echinocandin as first-line therapy, set out in the candidiasis guideline by Pappas et al. [22], and it carries an epidemiological lesson. Empirical antifungal defaults have to be triangulated against local species-level and susceptibility-level data that are kept current. Where fluconazole susceptibility cannot be assumed to be universal, such as in patients with previous azole exposure or in geographies with documented rising azole resistance, an echinocandin remains the appropriate empirical choice pending susceptibility results. The finding also makes a practical case for routine rather than exceptional in vitro susceptibility testing.

Zero candidemia mortality and universal source control

A notable outcome finding is the complete absence of candidemia-attributable mortality, despite fungemia in all 20 *Candida* episodes and a high proportion of underlying haemato-lymphoid malignancy. Contemporary paediatric candidemia mortality is variably reported at roughly 10-30%, with 10% 30-day mortality in the Fisher et al. cohort of 285 children [28] and 28.6% 30-day mortality across 248 episodes in the Karadag-Oncel et al. series [27]. The favourable outcome here went alongside prompt susceptibility-guided fluconazole therapy and removal of the CVAD in every one of the 10 patients with candidemia. With only 10 patients, we cannot interpret that association causally. Marrow recovery, underlying disease status and other factors are uncontrolled confounders, and the finding should be treated as hypothesis-generating. Zaoutis et al. [26], Karadag-Oncel et al. [27] and Fisher et al. [28] have shown, in successive paediatric candidemia cohorts, that a retained central venous catheter is independently associated with disseminated candidiasis and with death, and our data are consistent with the mortality benefit of aggressive source control in this population.

Invasive aspergillosis and its poor outcomes during intensive chemotherapy

Ten patients had invasive aspergillosis, four probable and six possible, and three of them (30%) died. Two of the three deaths were in the probable group and one in the possible group, so restricting the analysis to proven and probable disease would still have retained most of the mould-attributable mortality. We kept the possible cases for descriptive completeness, in line with the EORTC/MSGERC framework. Every fatal case was in a patient receiving intensive myelosuppressive chemotherapy at the time of diagnosis. This mortality figure is concordant with outcomes reported in intensively treated paediatric haematologic-malignancy cohorts, including a crude IFI mortality of 33.3% in the 16-year Kazakou et al. series [6] and a treatment success rate of only 33% among children with haematological malignancy in the paediatric voriconazole experience of Walsh et al. [25]. Kobayashi et al. add useful context, having found acute myeloid leukaemia, recurrent disease and older age to be independent predictors of IFI in children and adolescents with haematologic and malignant disease [30], as does the review of azole resistance and its effect on aspergillosis guidelines by Georgiadou and Kontoyiannis [31]. We used voriconazole first-line where clinically feasible, consistent with the landmark trial of Herbrecht et al., which demonstrated a 12-week survival advantage of voriconazole over amphotericin B [24], with the paediatric experience of Walsh et al. [25] and with the guideline recommendations of Warris et al. [5] and Patterson et al. [23]. Our 34.8% serum GM positivity reflects selective, suspicion-driven testing rather than assay sensitivity. Badiee et al. reported a serum GM sensitivity of 90% in a paediatric cohort [18], and Zhou et al. showed lavage GM to outperform serum GM in non-neutropenic invasive pulmonary aspergillosis [16], which together strengthen the case for lavage GM and imaging in diagnosing probable disease. The prevailing CT pattern in this cohort, a mix of consolidation at 15%, air-crescent sign at 11%, ground-glass opacity at 10% and nodular lesions at 7%, is congruent with the classical radiological signatures of paediatric invasive pulmonary aspergillosis and supports early CT triage in high-risk febrile neutropenia.

Mucormycosis and rare yeasts

Both patients with mucormycosis died, as did the single patient with *Geotrichum* (Magnusiomyces) *capitatum* infection, all three despite liposomal amphotericin B. Our mucormycosis outcomes are, if anything, worse than the 44-51% mortality reported in the St. Jude single-centre paediatric series of Marón et al. [32] and worse than the 46.7% overall mortality recorded across 388 proven or probable cases in the prospective Indian multicentre study of Prakash et al. [8]. The Indian context matters here, because Prakash and Chakrabarti put the prevalence of mucormycosis in India at roughly seventy times that in global data [10], which sits against the contemporary international epidemiology reviewed by Skiada et al. [9]. The *G. capitatum* death is congruent with the high mortality long reported for this rare yeast in acute leukaemia. Girmenia et al. reported death in 57.1% of 35 *G. capitatum* infections in an Italian multicentre haematology series [14], and Schuermans et al. documented fatal breakthrough *S. capitata* infection during echinocandin therapy [15]. Two things follow. Cure remains hard to achieve in mould disease of the profoundly neutropenic child, and the case for early biopsy-directed diagnosis, aggressive surgical debridement in mucormycosis and immune reconstitution wherever biologically feasible is correspondingly strong.

Diagnostic performance of biomarkers and BAL

Serum GM was the non-culture test we applied most consistently, being positive in 34.8% of patients overall (8 of 23). BDG and lavage were applied in only a minority of cases, which reflects resource and feasibility constraints rather than any doctrinal choice, a limitation the ESCMID-ECMM paediatric guidance itself acknowledges [5]. Where they were performed, BDG was positive in 2 of 3 patients, and lavage was positive in 1 of 2, contributing to case classification, consistent with the paediatric biomarker experience of Salvatore et al. [17] and the paediatric lavage yield reported by Efrati et al. [19] and Nadimpalli et al. [20]. Wider adoption of serial GM, BDG and directed lavage would probably improve early case ascertainment and shorten the time to targeted therapy.

Comparison with the published literature

Table 5 sets the present cohort beside contemporary PubMed-indexed paediatric and adult literature on incidence, species distribution, susceptibility and mortality. Our candidemia outcomes are favourable against published paediatric benchmarks, our mould mortality falls within the expected range for invasive aspergillosis and our mucormycosis outcomes are worse than expected in a two-patient sample. The data extracted are those explicitly reported in the cited publications.

**Table 5:** Benchmarking the present cohort against contemporary published literature. Comparator numbers are extracted verbatim from the cited PubMed-indexed publications. Statistical test note. This table presents a descriptive benchmarking of the present cohort against published literature. Comparator values are reproduced verbatim from the source publications, and no primary statistical comparison is performed by the present authors. No comparator p-values are reproduced here. Where a source publication reported an effect estimate, for example, the 12-week survival hazard ratio of 0.59 (95% CI 0.40 to 0.88), in Herbrecht et al. 2002, that estimate is the source publication's own. ALL: Acute lymphoblastic leukaemia; AML: Acute myeloid leukaemia; NHL: Non-Hodgkin lymphoma; FN: Febrile neutropenia; IA: Invasive aspergillosis; IFI: Invasive fungal infection.

Study (Year)	Design and setting	N	Key comparator finding	Present-study equivalent
Kazakou et al., 2020 [6]	Retrospective, Greek paediatric haemato-oncology, 16 years	297	IFI 10.5% (ALL), 18.2% (AML), 2.8% (NHL)	5.8% overall among febrile patients
Kumar et al., 2018 [7]	Prospective, AIIMS, Delhi, paediatric acute leukaemia with persistent febrile neutropenia	74	IFI prevalence 22.97%	43.5% breakthrough IFI on prophylaxis
Xess et al., 2007 [11]	AIIMS, Delhi, five-year candidemia surveillance	275	*C. tropicalis* (35.3%), *C. albicans* (21.5%), *C. parapsilosis* (20%)	*C. tropicalis* (60%), *C. parapsilosis* (20%), *C. albicans* (15%)
Dewan et al., 2015 [13]	Indian tertiary-care centre, candidemia in haematological malignancy	150 screened, 15 candidemic	*C. tropicalis* (46.7%) of isolates, non-albicans 2.75:1	Non-albicans (85%), *C. tropicalis* (60%)
Herbrecht et al., 2002 [24]	Multinational randomised controlled trial, invasive aspergillosis in adults and adolescents	277	12-week survival of 70.8% with voriconazole versus 57.9% with amphotericin B	Invasive aspergillosis mortality (30%), with voriconazole first-line where feasible
Marón et al., 2024 [32]	St. Jude single-centre paediatric mucormycosis, 1970-2019	44	Mortality (44-51%), Rhizopus (40%), prolonged neutropenia (91%)	Mucormycosis mortality (100%), n = 2
Girmenia et al., 2005 [14]	Italian multicentre, *Geotrichum* and *Trichosporon* in haematological malignancy	52	*G. capitatum* in acute myeloid leukaemia, death in 57.1%	Geotrichum mortality (100%), n = 1
Cornely et al., 2007 [21]	Randomised controlled trial, posaconazole prophylaxis in neutropenia from acute myeloid leukaemia or myelodysplastic syndrome	602	Proven or probable IFI 2% with posaconazole versus 8% with control	Breakthrough on prophylaxis in 10 of 23 (43.5%)

Clinical implications

Four clinical implications follow from these data. First, the local dominance of *C. tropicalis,* together with universal fluconazole susceptibility, supports a susceptibility-guided, fluconazole-based first-line strategy for paediatric candidemia at this centre. Institutions without documented universal susceptibility should retain an echinocandin as the empirical default, in line with the ECIL [3] and IDSA [22] guidance. Second, aggressive device management, including the universal early removal achieved here, is a strong modifiable determinant of mortality and should be treated as a non-negotiable part of source-control bundles for candidemia, based on the evidence of Zaoutis et al. [26], Karadag-Oncel et al. [27] and Fisher et al. [28]. Third, in high-risk children with persistent febrile neutropenia, prolonged mucosal breakdown or clinical sinusitis, the very high mucormycosis mortality we observed, read against the elevated Indian burden documented by Prakash et al. [8], Skiada et al. [9], Prakash and Chakrabarti [10] and Marón et al. [32], argues for early cross-sectional imaging, tissue biopsy and prompt liposomal amphotericin B pending confirmation. Fourth, structured integration of serum GM, BDG and lavage, consistent with the EORTC/MSGERC 2020 criteria [2] and the guidance of Warris et al. [5] and Patterson et al. [23], has real potential to bring diagnosis forward and improve outcomes.

Limitations and future directions

The limitations of this study are those of a retrospective single-centre design. The modest sample size precludes multivariable analysis of mortality determinants, and the pathogen-specific outcomes for mucormycosis and *Geotrichum* rest on very small numerators. Serum BDG and lavage were performed in only a minority of patients, which limits what we can say about their real-world performance here. Antifungal serum drug levels were not systematically retrievable across all episodes, and susceptibility was interpreted categorically because we did not use quantitative minimum inhibitory concentration values. Serum GM was measured on single samples rather than serially, so a rising-titre pattern could not be used to support case classification. Therapeutic drug monitoring of mould-active azoles was not performed, so we cannot separate a breakthrough episode from subtherapeutic drug exposure or incomplete adherence. Six of the ten aspergillosis patients met possible rather than probable criteria. These cases are labelled separately wherever aspergillosis is discussed, but their inclusion for descriptive completeness does mean the aspergillosis figures are not confined to proven and probable disease. Our primary denominator is the 396 unique febrile paediatric haemato-oncology patients screened over the study period, a febrile rather than a whole-unit denominator. The figure we report is therefore a cumulative incidence among febrile patients and not a true at-risk cohort incidence, and the cross-cohort comparisons in Table 5 should be read with that difference in mind. Earlier drafts mixed patient-level and episode-level denominators. The patient is now the primary unit of analysis throughout, and episode counts are labelled as such wherever they appear. Finally, follow-up was limited to the index episode, and longer-term neurodevelopmental and quality-of-life outcomes in survivors merit prospective evaluation. Three priorities follow. Multicentre paediatric IFI surveillance across Indian centres would define a national epidemiological and susceptibility benchmark. Prospective biomarker-driven diagnostic algorithms incorporating serial GM, BDG and imaging need testing. Formal cost-effectiveness evaluation of universal mould-active prophylaxis in the Indian paediatric AML and transplant setting is overdue.

## Conclusions

In this five-year South Indian paediatric haemato-oncology cohort, invasive fungal disease was documented in 5.8% of febrile patients. *Candida* and *Aspergillus* co-dominated, with *C. tropicalis* predominating among candidaemic isolates. Every *Candida* isolate was fluconazole-susceptible, which allowed a fluconazole-based first-line strategy. The CVAD was removed in every patient with candidemia, and no candidemia-attributable death occurred. Both patients with mucormycosis and the single patient with *Geotrichum* infection died, although the numbers were too small to support a firm inference. Crude overall mortality was 26.1%, driven entirely by mould and rare-yeast disease. These data support locally informed, susceptibility-guided first-line antifungal selection; aggressive device-based source control for candidemia; vigilant surveillance for mucormycosis in high-risk children given the Indian epidemiological setting; and structured use of serum GM, BDG and directed BAL in line with the revised consensus criteria. Multicentre Indian paediatric surveillance is the priority if national benchmarks are to inform empirical practice.
